# Biotechnology and the Future of Vaccines—From Novel Routes and Vectors to Safety, Efficacy, and Global Impact

**DOI:** 10.3390/vaccines13101043

**Published:** 2025-10-10

**Authors:** Tsu-Hsiang Kuo, Yuan-Chuan Chen

**Affiliations:** 1 Department of Rehabilitation Science, Jenteh Junior College of Medicine, Nursing and Management, Houlong Township, Miaoli County 35664, Taiwan; dennissylvie@gmail.com; 2 Department of Medical Technology, Jenteh Junior College of Medicine, Nursing and Management, Houlong Township, Miaoli County 35664, Taiwan; 3 Department of Nursing, Jenteh Junior College of Medicine, Nursing and Management, Houlong Township, Miaoli County 35664, Taiwan

## 1. Introduction: Vaccines in a Transformative Era

Vaccines remain one of the greatest achievements in biomedical science, credited with the eradication of smallpox, the near-elimination of polio and the prevention of many deaths from infectious diseases. However, as the world confronts emerging pandemics and persistent threats such as rabies, cytomegalovirus and the rising burden of non-communicable diseases such as cancer, the limitations of traditional vaccine platforms are increasingly apparent. Classical formulations including inactivated, live attenuated and subunit vaccines delivered predominantly by oral, intramuscular (IM), or subcutaneous (SC) routes have saved millions of lives. However, they are constrained by production timelines, safety concerns, stability issues, and limited adaptability to novel pathogens.

The past two decades, accelerated by the COVID-19 pandemic, have witnessed a revolution in vaccine science driven by biotechnology. Recombinant proteins, DNA vaccines, mRNA vaccines, nanoparticle-based formulations, microneedle patches, and inhalable aerosols are now at the forefront of translational research. The convergence of nanotechnology, polymer chemistry, genetic engineering, and immunology is enabling the development of vaccines that are faster to design, easier to administer, and capable of addressing both infectious and non-infectious diseases.

The six articles summarized in this Special Issue, “Biotechnologies Applied in Vaccine Research”, provide a panoramic view of this landscape, from the design of lipid nanoparticles (LNPs) for mRNA delivery to polymeric micelles enhancing endosomal escape; from process optimization in Vero cell-based rabies vaccine manufacturing to *Salmonella*-based oral vaccines; and from novel administration routes to next-generation biotechnologies. These contributions collectively illuminate both the promise and the challenges of next-generation vaccine science.

This editorial synthesizes their key findings and situates them within the broader trajectory of global vaccine development. We believe that biotechnology is not only providing incremental improvements but is fundamentally redefining what vaccines are, how they are delivered, and what diseases they can target.

## 2. New Frontiers in Administration Routes

Traditional vaccines are largely administered orally, intramuscularly or subcutaneously. While effective, these methods have limitations: needle phobia reduces compliance, cold-chain requirements limit distribution, and systemic delivery often induces suboptimal mucosal immunity.

As highlighted in the paper “Novel Administration Routes, Delivery Vectors, and Application of Vaccines Based on Biotechnologies: A Review” [1], recent advances are challenging this paradigm. Microneedles, inhalable aerosols and transcutaneous delivery platforms are emerging as viable alternatives. Microneedle patches, for instance, deliver antigens painlessly into the dermis, a tissue rich in antigen-presenting cells, thereby enhancing immunogenicity while improving patient acceptance. Inhaled vaccines can induce mucosal immunity in the respiratory tract, offering advantages against airborne pathogens like *Mycobacterium tuberculosis*, influenza virus, respiratory syncytial virus (RSV), and coronaviruses.

These innovations are not mere conveniences; they represent strategic tools to expand vaccine coverage, particularly in low-resource settings where cold-chain infrastructure and trained personnel are limited. If vaccines are widely adopted, alternative administration routes could democratize access and transform delivery from a hospital-based intervention into a community-based or even self-administered public health strategy.

## 3. Lipid Nanoparticles: The Double-Edged Sword of mRNA Delivery

The success of mRNA vaccines against COVID-19 showcased the enormous potential of nucleic acid-based therapeutics. At the heart of this breakthrough lies LNP technology. Composed of ionizable lipids, phospholipids, cholesterol, and polyethylene glycol (PEG) lipids, LNPs protect fragile mRNA molecules from degradation to facilitate cellular uptake and enable endosomal release.

The article “Recent Advances in Lipid Nanoparticles and Their Safety Concerns for mRNA Delivery” [2] underscores that while LNPs have revolutionized mRNA delivery, risk and toxicity, reactogenicity, and immunogenicity are still central concerns. For instance, PEG lipids have been linked to hypersensitivity reactions, and accumulation of synthetic lipids may trigger inflammatory responses or hepatic stress. Furthermore, variability in biodistribution and clearance complicates long-term safety assessments.

This duality—efficacy on one hand, safety concerns on the other—reflects the central challenge of next-generation vaccines. The field must balance rapid innovation with rigorous toxicological evaluation. Some specific strategies such as biodegradable lipid analogs, targeted delivery systems and personalized dosing regimens may mitigate risks while preserving efficacy. Importantly, regulatory frameworks must evolve to assess these complex, dynamic formulations without stifling innovation.

## 4. Ensuring Safety and Quality: Lessons from Rabies Vaccines

While futuristic platforms dominate headlines, traditional vaccines remain indispensable. Rabies, a fatal but preventable disease, continues to demand safe and effective vaccines, especially in Asia and Africa. The paper “Evaluation of the Efficacy of the Vaccine Production Process in Removing Residual Host Cell DNA from the Vero Cell Rabies Vaccine” [3] provides a case study on the importance of process optimization and quality control.

Vero cells, widely used for rabies vaccine production, pose a theoretical risk due to residual host cell DNA (HCD). The study demonstrated that a carefully optimized production pipeline—incorporating multiple purification steps, qPCR and electrophoresis monitoring, and enzyme-linked immunosorbent assay (ELISA)-based antigen quantification—can achieve antigen recovery rates above 8.5% and HCD removal rates exceeding 99.99%. The residual DNA levels were well below the World Health Organization (WHO) threshold of 2 ng/dose, and fragments were predominantly under 200 base pairs, further minimizing oncogenic risk.

This case exemplifies the often-overlooked reality that innovation must go hand-in-hand with meticulous quality assurance. As new vaccine platforms proliferate, robust methods for monitoring impurities, residual materials, and batch-to-batch consistency will be critical for public trust and regulatory acceptance.

## 5. Beyond Lipids: Polymeric Micelles and the Quest for Endosomal Escape

One of the bottlenecks in nucleic acid-based therapeutics is endosomal entrapment. After cellular uptake, LNPs often fail to release their mRNA payload into the cytoplasm, limiting translation efficiency. An elegant solution is presented in the paper “Development of a Cationic Polymeric Micellar Structure with Endosomal Escape Capability Enables Enhanced Intramuscular Transfection of mRNA-LNPs” [4].

The authors engineered a cationic polymeric micelle that synergizes with conventional LNPs to destabilize endosomal membranes and promote cytosolic release. In vivo experiments with firefly luciferase mRNA demonstrated a two- to three-fold increase in protein expression compared to standard LNPs. Importantly, this was achieved without altering the inherent properties of the LNPs themselves, thereby preserving formulation stability and manufacturability.

This innovation highlights the modular nature of biotechnology: new materials can be layered onto existing platforms to overcome specific biological barriers. Polymeric micelles may not replace LNPs but rather complement them, paving the way for hybrid delivery systems that maximize efficacy while minimizing risk.

## 6. Living Vectors: Harnessing *Salmonella* for Oral Vaccination

Not all innovations rely on synthetic nanomaterials. The paper “Effective Immune Protection of Mice from Murine Cytomegalovirus Infection by Oral *Salmonella*-Based Vaccine Expressing Viral M78 Antigen” [5] explores the use of attenuated bacteria as oral vaccine vectors. By engineering *Salmonella* to express the M78 antigen of murine cytomegalovirus (MCMV), the authors demonstrated induction of both humoral and cellular immunity in mice, including serum IgG, mucosal IgA, and T-cell responses. Vaccinated mice were protected against both intraperitoneal and intranasal MCMV challenges, with significantly reduced viral titers in multiple organs.

This approach carries profound implications. Oral *Salmonella*-based vaccines could be particularly transformative in low-resource settings, eliminating the need for cold chains, sterile injections, and highly trained personnel. Moreover, they leverage the mucosal immune system which is often neglected by injectable vaccines to provide first-line defense against pathogens entering via the respiratory or gastrointestinal tracts.

Challenges remain, including ensuring safety in immunocompromised individuals, controlling bacterial persistence, and avoiding interference from gut microbiota. However, as proof of concept, this study positions bacterial vectors as a promising alternative in the expanding vaccine toolkit.

## 7. Biotechnology and the Expanding Scope of Vaccines

The article “Advances in Biotechnology and the Development of Novel Human Vaccines” [6] provides a sweeping overview of how biotechnology is expanding the scope of vaccine applications. Beyond infectious diseases, vaccines are now being designed against cancers, allergies, and even metabolic disorders. Platforms such as virus-like particles (VLPs), bacteriophage-based vectors and next-generation adjuvants targeting innate immune receptors are enabling unprecedented flexibility in antigen design and immune modulation.

Moreover, alternative delivery routes including transcutaneous patches, mucosal sprays and microneedles promise to overcome logistical barriers, while adjuvant innovations aim to enhance responses in vulnerable populations such as the elderly, neonates and immunocompromised patients.

In short, biotechnology is not only redefining how vaccines are made but also what diseases they can target and who can benefit from vaccination.

## 8. Ethical, Regulatory, and Global Considerations

While scientific innovation accelerates, several cross-cutting issues demand attention.

(1) Equity and Access: Novel vaccines are usually expensive and technologically demanding to produce. Without intentional strategies for equitable distribution, biotech revolution in vaccine development may exacerbate global health disparities.
(2) Regulatory Adaptation: Traditional frameworks built around protein or inactivated-virus vaccines may be ill-suited to assess dynamic nanomaterials, synthetic polymers, and living vectors. Regulators must evolve without compromising safety.
(3) Public Trust: Vaccine hesitancy, amplified during the COVID-19 pandemic, underscores the need for transparency in communicating both benefits and risks. Quality control such as that demonstrated in the rabies vaccine study is crucial for sustaining confidence.
(4) Long-Term Safety: As highlighted in LNP safety studies, the long-term consequences of repeated exposure to novel materials remain unclear. Continuous post-marketing surveillance will be essential.

## 9. Conclusions: Toward a New Vaccine Era

Taken together, the six papers in this Special Issue illustrate the multifaceted revolution underway in vaccine science. Novel administration routes promise broader acceptance; LNPs and polymeric micelles unlock the potential of mRNA; quality assurance in classical vaccines safeguards trust; bacterial vectors offer oral delivery alternatives; and biotechnology as a whole expands the horizons of what vaccines can achieve.

It is clear that vaccines are becoming more personalized, more adaptable, and more integrated with cutting-edge biotechnology. Despite this, challenges in safety, regulation, equity, and public trust remain. The scientific community, policymakers and industry must work collaboratively to ensure that the benefits of these revolutionary vaccines are realized globally and responsibly. The 21st century may well be remembered as the “vaccine century.” But whether it fulfills this promise depends not merely on technological breakthroughs but also on our collective commitment to ensure that vaccines are safe, accessible, and trusted by most people.
